# I Plan Therefore I Choose: Free-Choice Bias Due to Prior Action-Probability but Not Action-Value

**DOI:** 10.3389/fnbeh.2015.00315

**Published:** 2015-11-25

**Authors:** Lalitta Suriya-Arunroj, Alexander Gail

**Affiliations:** ^1^Sensorimotor Group, German Primate CenterGöttingen, Germany; ^2^Bernstein Center for Computational NeuroscienceGöttingen, Germany; ^3^Faculty of Biology and Psychology, Georg-Elias-Müller Institute, Georg August UniversityGöttingen, Germany

**Keywords:** reach movement, decision-making, action selection, motor planning, bias, prior probability, expected value

## Abstract

According to an emerging view, decision-making, and motor planning are tightly entangled at the level of neural processing. Choice is influenced not only by the values associated with different options, but also biased by other factors. Here we test the hypothesis that preliminary action planning can induce choice biases gradually and independently of objective value when planning overlaps with one of the potential action alternatives. Subjects performed center-out reaches obeying either a clockwise or counterclockwise cue-response rule in two tasks. In the probabilistic task, a pre-cue indicated the probability of each of the two potential rules to become valid. When the subsequent rule-cue unambiguously indicated which of the pre-cued rules was actually valid (instructed trials), subjects responded faster to rules pre-cued with higher probability. When subjects were allowed to choose freely between two equally rewarded rules (choice trials) they chose the originally more likely rule more often and faster, despite the lack of an objective advantage in selecting this target. In the amount task, the pre-cue indicated the amount of potential reward associated with each rule. Subjects responded faster to rules pre-cued with higher reward amount in instructed trials of the amount task, equivalent to the more likely rule in the probabilistic task. Yet, in contrast, subjects showed hardly any choice bias and no increase in response speed in favor of the original high-reward target in the choice trials of the amount task. We conclude that free-choice behavior is robustly biased when predictability encourages the planning of one of the potential responses, while prior reward expectations without action planning do not induce such strong bias. Our results provide behavioral evidence for distinct contributions of expected value and action planning in decision-making and a tight interdependence of motor planning and action selection, supporting the idea that the underlying neural mechanisms overlap.

## Introduction

During economic choice, we weigh potential options. In general, the most beneficial or least costly option directs our decision. When deciding between equally valued options, an economic decision results in choice of either option with equal probability, known as matching behavior (Herrnstein, 1961; Pierce and Epling, 1983; Sugrue et al., 2004; Lau and Glimcher, 2005). However, factors other than value-based preference can also influence our choices. In statistical decision theory, value is one building block, which together with prior probability and evidence, forms the three main computational elements of decision-making processes (Gold and Shadlen, 2007). Furthermore, choices normally require action selection. Independent of the expected reward, differences in physical effort between response options can be an obvious motor-related decision factor (Kurniawan et al., 2011; Rigoux and Guigon, 2012; Burk et al., 2014). But more than that, covert motor-related factors, like prior action planning, could also be expected to influence later overt responses, and hence decision outcomes (Cisek, 2007; Gallivan et al., 2015). Here we compare the effect of prior motor planning with prior value-based preference on choice probabilities and choice reaction times in situations with balanced reward (neutral free choice) at the time of the actual decision.

Imagine you are late for dinner. Your two alternative routes home are on average equally fast but depend on current traffic. In scenario 1, the slow truck in front of you indicates a turn to the right. This will likely make you plan a left turn at the next intersection, because with the truck going right, the left route now is faster, hence has a higher value. If the truck then unexpectedly pulls into a rest area you are free to choose between equal-valued options (neutral choice) once you arrive at the intersection (time of commitment). Will you stick with your prior plan, even though both alternatives would now be equally attractive? In scenario 2, the truck's indicators are so dirty that you cannot identify an indicated direction, but you know that the left route would allow you to overtake the truck more easily than the right route. This means the left route has a higher (truck-conditional) value, as the truck would slow you down less. If the truck then unexpectedly pulls into a rest area, both options are equal-valued again. Will you decide based on your prior value-based preference? Both scenarios have in common that the value is initially higher for the left route, in scenario 1 due to an imbalance in prior probability, in scenario 2 due to an imbalance in prior value of a decision-critical event that is yet to happen (the truck's actual turn = evidence). Also, in both scenarios, you will be provided with evidence immediately before your commitment, and most times (truck turns left or right) this will directly instruct your choice (go the other way). Finally, in both scenarios, the expected value becomes neutralized when the truck pulls into the rest area, rendering both of your alternatives equally valued. The scenarios differ in their intuitive effect on action planning. In scenario 1 your initial preference based on the probability of the later event encourages you to preliminarily plan your action. Instead, in scenario 2, even though you also have an initial value-based preference, the situation discourages specific action planning until you know the truck's actual turn, since each turn is equally likely. Will an *a priori* value-based preference in which action planning is discouraged affect your later neutral choice differently to an *a priori* preference that is associated with an action plan? This is the question we address in this study.

To investigate the effect of motor planning on choice, we need to dissociate planning from value-based preference. It is known that movement planning is encouraged by motor-goal predictability. For example, tasks with probabilistic pre-cues, as used in early attention studies (Posner et al., 1980), have been adopted in sensorimotor studies to test the ability to plan movements in Parkinson's disease patients (Stelmach et al., 1986; Jahanshahi et al., 1992; Praamstra et al., 1996; Leis et al., 2005). In such tasks, the pre-cue correctly indicates the location of an upcoming target with a typical probability of 80% (cue validity) while in the remaining 20% of trials the non-cued target will be instructed. A subsequent imperative cue instructs the subject when and toward which target location to act. The prior information contained in the pre-cue encourages subjects to plan the movement toward the pre-cued target, confirmed by effects of cue-validity on reaction times and, in some cases, movement times (Leis et al., 2005). Following the rationale of cue validity, a neutral pre-cue indicating equal probability of occurrence for each target should not evoke imbalanced preliminary planning. We used this rationale for testing the biasing effect of motor planning on reward-balanced choices. For this we manipulated the degree of motor planning by different degrees of *motor-goal predictability*.

However, probabilistic pre-cues can confound predictability with *preferability of a motor goal*. Among multiple targets, if the validity of one target becomes larger, the probability of receiving reward at that target also increases, and hence the expected value, defined as the product of probability and amount of reward (Von Neumann and Morgenstern, 1944; Gold and Shadlen, 2007; Levy and Glimcher, 2012), will also increase for the higher-validity target. In order to disentangle the effect of planning from the effect of reward expectation, we designed two tasks with matched expected rewards but only one of which encouraged preliminary action planning.

In the context of probabilistic choice behavior, a prior should have more impact when evidence is weaker (Körding and Wolpert, 2004; Vilares et al., 2012). Therefore, the effect of the prior is typically investigated in situations when decisions are based on ambiguous evidence. For example, using random dot motion stimuli, priors were shown to influence the interpretation of ambiguous visual sensory evidence (e.g., Mulder et al., 2012) and affect the latency of action initiation (e.g., Carpenter and Williams, 1995). Yet, if the perceptual interpretation of the evidence is one-to-one associated with a behavioral response then a prior is also likely to invoke preliminary action planning. It can therefore be difficult to disentangle whether the effect of the prior on choice is mediated via an effect on sensory processing or on action planning. Here we test the effect of prior probabilities without any perceptual uncertainty, emphasizing the effect of action planning on choice.

Even though our experiment did not utilize ambiguous cumulative sensory evidence toward a perceptual decision, but rather immediate unambiguous evidence (instructed trials) or rule-neutral evidence (choice trials), we find it helpful to conceptualize our study in the context of drift-diffusion models (DDM). On the basis of the DDM, conceptualizing decision processes as a gradual accumulation of evidence toward one of two alternative boundaries (e.g., Ratcliff, 1978; Ratcliff et al., 1999), bias can be explained by different computational mechanisms: (1) a shorter migration distance, either due to a baseline shift (Ratcliff, 1985) or a bound shift (Ratcliff, 1978), or (2) a change in drift rate (Ratcliff, 1981). These mechanisms allow accumulated evidence to reach one bound with smaller reaction times (RT) and higher choice probabilities (CP). Which mechanism is responsible for RT reduction and CP increase in which behavioral context is a topic of ongoing research (Summerfield and Tsetsos, 2012). In the context of perceptual decision-making, previous studies showed that prior probability adapts migration distance (Bogacz et al., 2006; Simen et al., 2009; Mulder et al., 2012) while strength of evidence steers drift rate (Roitman and Shadlen, 2002; Coallier and Kalaska, 2014; Coallier et al., 2015; Hanks et al., 2015). However, the effect of expected value as been accounted for by different explanations: baseline shift (Maddox, 2002; Bogacz et al., 2006; Mulder et al., 2012) or drift rate change (Diederich and Busemeyer, 2006). The diversity of explanations for the effect of expected value could be due to insufficient systematic dissociation of other factors from the effects of movement planning as previous studies usually involved probabilistic choice tasks, which we avoid here. Additionally, we test the specific hypothesis that preference leads to the same biasing effects as planning, except for a downscaling factor that reduces effect strength (Maddox and Bohil, 1998; Bogacz, 2007; Mulder et al., 2012). Such downscaling should be particularly obvious when testing multiple levels of bias, in which case it should be possible to estimate the value-based bias from the probabilistic bias by applying a fixed gain factor. Instead of using only a single level of bias manipulation, we therefore probed for a graded effect of graded prior probability as opposed to graded value.

To our knowledge, no study has directly tested the proportional effect of action planning on choices in which there is no difference in expected value between options. According to emerging evidence, neural mechanisms overlap between decision-making and movement planning (Cisek, 2007; Scherberger and Andersen, 2007; Lindner et al., 2010; Klaes et al., 2011; Coallier et al., 2015; Gallivan et al., 2015; Hanks et al., 2015). We therefore hypothesize that previously planned actions should bias later neutral choices in favor of these actions, independently of reward expectation.

## Materials and methods

### Participants

Forty-three subjects (30 females, age (mean ± SD): 27.45 ± 4.89) participated in the study as paid volunteers. Among the 43 subjects, 31 participated in both AMNT and PROB tasks (on 2 separate days; 19 did PROB task first and 12 did AMNT task first), 10 in only PROB task, and 2 in only AMNT task. All subjects were healthy, right-handed, and had self-reported normal or corrected-to-normal vision. Detailed written instructions were given to the subjects before the experiment. Prior to each recording session, subjects were familiarized with the set-up and practiced the task. All subjects gave written informed consent for participation. Experiments were in accordance with institutional guidelines for experiments with humans and adhered to the principles of the Declaration of Helsinki. The experimental protocol was approved by the ethics committee of the Georg-Elias-Mueller-Institute for Psychology, University of Goettingen.

We included all 43 subjects in the analyses. When comparing among conditions within each task with *post-hoc* tests, we included the 33 subjects who participated in the AMNT task and 41 subjects who participated in the PROB task. When comparing between tasks with *post-hoc* tests, we included the 31 subjects who participated in both tasks.

### Rule-selection task with sequential cueing

**Figure 1:**
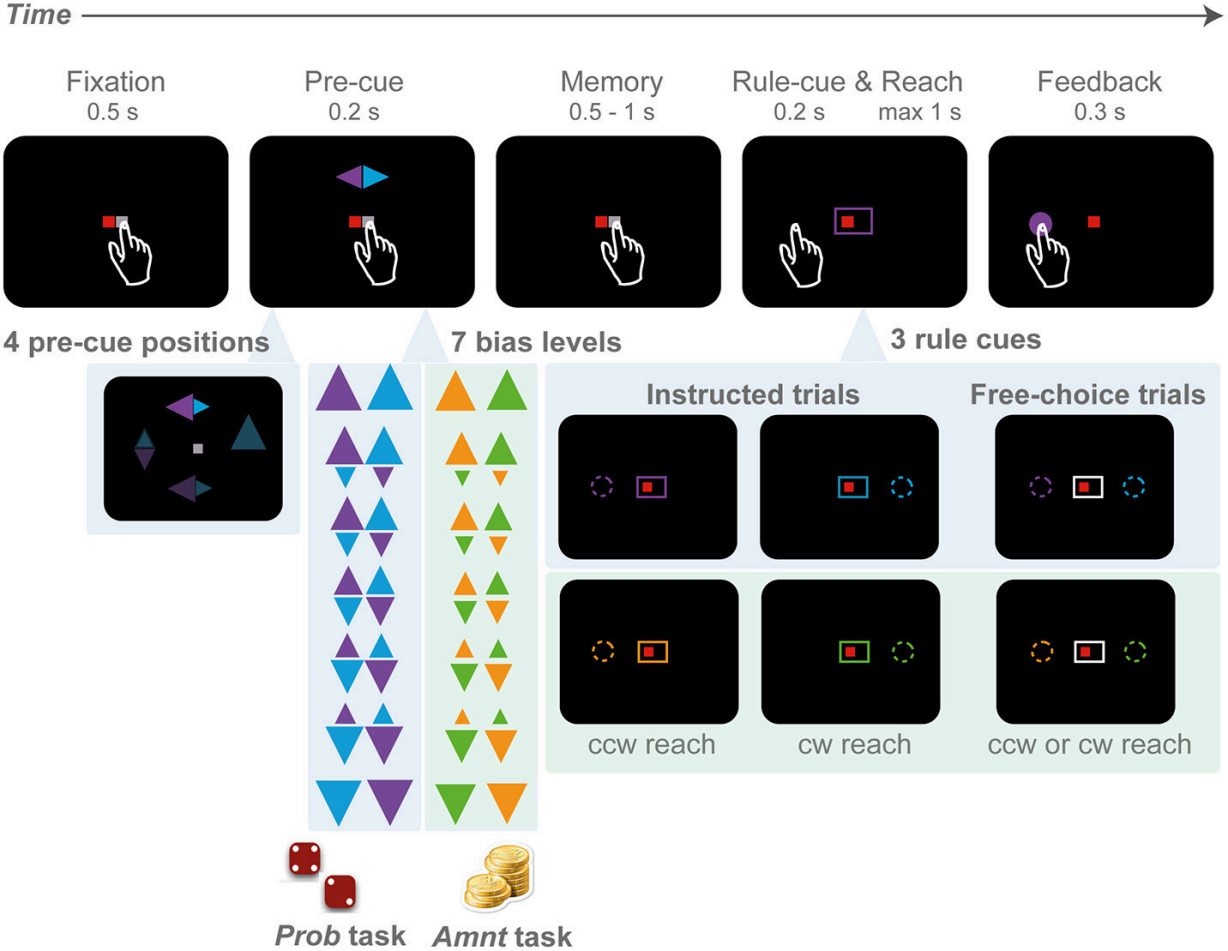
**Rule-selection tasks with probabilistic (PROB) and amount (AMNT) bias manipulations**. A trial starts with eye-hand fixation. Next, a pre-cue appears at one of the four cardinal locations (top, down, left, right), indicating two potential goals and the probability of each goal in the PROB task (big triangle - high probability), or the reward amount associated with each goal in the AMNT task (big triangle - more reward). In the PROB task, the reward amount is kept constant in all conditions, whereas in the AMNT task the probability is kept equal in all conditions. After a memory period, the rule-cue appears and either indicates one valid target or leaves the options open so that subjects can freely choose. Both options in choice trials always provide equal value.

The idea of the study was to investigate the influence of rule predictability and pure preference on choice behavior. The task implements the idea of the traffic example in the introduction. We designed a center-out reach task with sequential cueing. A first pre-cue raised expectations on either the probability or the value of a later rule instruction (Figure 1). The rule instruction (rule-cue) provided final information on the actual rule and hence the action(s) to be rewarded. Two potential reach goals had to be inferred from the single pre-cued location based on clockwise (*cw*) and counterclockwise (*ccw*) transformation rules. We implemented two variants of this rule selection task, one in which the prior probability of either rule to be instructed was announced in advance by the pre-cue (PROB task), another in which the reward of either rule, in case it would be instructed, was announced (AMNT task). The choice experiment was risk-free, since at the time of the required behavioral response (decision), there was no uncertainty about the outcome; subjects were either instructed about the correct response immediately before the decision (instructed trials = truck turns left or right in the example from the Introduction), or they were free to choose among both options with 100% reward probability and equal reward amount for each option (choice trials = truck pulls into rest area). Note that the pre-cue was only informative about the reward structure of the instructed trials, while free-choice options were safe and equal-valued. As a consequence, subjects could achieve 100% reward probability with proper performance in all task conditions. The reward delivered in each trial was accumulated and translated into the compensation that participants received at the end of the session (see below).

Due to the temporal separation between pre-cue and rule-cue, each choice was preceded by a brief planning period (approaching the intersection in the truck example). During the planning period subjects were uncertain about the type of trial (instructed or choice), and uncertain about what the instruction will be. For optimal performance, subjects in response to the final instruction had to either follow their initial expectation or countermand it (instructed trials), or freely choose (choice trials). For example, a pre-cue in the AMNT task might raise the initial expectation that a left-side reach would be preferable, but the rule cue at the end of the planning period could still indicate a left-side or right-side single correct option (instructed trials) or two correct options (choice trial). The pre-cue could not be ignored, though, since the rule-cue only was meaningful in relation to the pre-cue.

The idea of this task design was that with a majority of instructed trials the pre-cue would induce a trial-by-trial behavioral bias, either based on *predictability* or *preferability*. In a first step, we had to confirm that both manipulations were effective in by analyzing subjects' responses in the instructed trials. In instructed trials the manipulations had an actual effect on the reward outcome, hence an effect on behavior had to be expected. As the main research question, we test if the variable predictability and preferability had the same effects on free-choice behavior. We probed this with randomly interspersed choice trials. Since the choice trials were value-balanced and risk-free, any choice probabilities and other behavioral biases should reflect the subject's *a priori* bias induced by the predictability or preferability resulting from the pre-cue, since no further immediate evidence supporting either rule is provided during the remainder of a choice trial.

Importantly, in the AMNT task, we aimed to induce a preference in the subjects without encouraging planning of the according action since either rule was equally likely to be instructed. On the other hand, the PROB task instead would encourage preliminary planning of the action that was associated with the most likely correct option since, in the likely case of an instructed trial, the instruction would match the rule expectation. We tested the assumption that predictability, as opposed to preferability, would lead to target-specific motor planning by additionally analyzing movement execution parameters in instructed trials. If such test yielded evidence for motor planning in predictable but not in preferable trials then any effects of predictability on choice behavior could be explained by an effect of motor planning.

We then compared the effect on risk-free choice behavior of either a preliminary value-based target preference (henceforth referred to as “preferability,” AMNT experiment) or a preliminary motor plan toward a probable target (henceforth referred to as “predictability,” PROB experiment). We will first describe in detail the elements of the task that are common to both experiments and then the differences in the reward schedule of the two experiments.

### Graded bias manipulation in the rule-selection tasks

Subjects in both experiments had to choose between either a clockwise (*cw*) or counter-clockwise (*ccw*) spatial mapping rule to infer the reach target relative to the position of the pre-cue (rule-selection task). Subjects were requested to perform reaches from the center to one of the four cardinal (0°, 90°, 180°, and 270°) targets in the periphery (center-out reach) on a touch screen (eccentricity of 9 cm with ~40 cm of screen distance, depending on subjects' reaching range), while maintaining gaze at the screen center (eye fixation) throughout the trial. The target locations had to be inferred from an incongruent cue location and were not marked by visual stimuli (rule-based movement).

Each trial started with a fixation period. Small red and white squares were presented at the center of the screen as eye and hand fixation points, respectively (Figure 1). Subjects initiated the trial by directing gaze to the eye fixation spot and, at the same time, touching the hand fixation spot (tolerance window: 3 cm radius). After a random period of 500–1000 ms fixation, a spatial pre-cue flashed briefly (200 ms) at one of the four cardinal target positions. The pre-cue consisted of two differently colored triangles, one pointing to the *cw*, the other to the *ccw* direction. The two triangles indicated the only two possible reach targets in a given trial, one at 90° *cw* and one at 90° *ccw* from the pre-cue, at identical eccentricity.

There was no fixed association between rules (*cw* or *ccw*) and colors. Subjects had to remember the pre-cue colors and match these with the subsequent color information of the rule-cue to complete the trial correctly. At the end of the following random-length memory period (500–1000 ms), the rule-cue appeared. The rule-cue consisted of a box framing the fixation points and was either colored to match one of the pre-cue triangles (instructed trials), or color-neutral (white; choice trials). In the instructed trials the colored rule-cue narrowed down the two potential targets to only one single correct target (*cw* or *ccw*). The reward probability for the instructed target was always 100%, for the alternative target 0%. In the choice trials, both potential targets indicated by the pre-cue were rendered valid with 100% reward probability by the color-neutral rule-cue. In choice trials the reward amount was always fixed and independent of the size of the pre-cue or any previous choice responses (reward-all schedule). Simultaneously with the onset of the rule-cue, the hand fixation point disappeared (“go” signal) and subjects had to reach toward the instructed or chosen target within a maximum of 1000 ms. In each block of six trials, two trials were randomly set to be choice trials, and the remaining four were instructed trials. Each unsuccessful trial was reinserted into the trial sequence.

In case of successful acquisition of a rewarded target, subjects received positive feedback in the form of a circular patch at the target position with an encouraging high-pitched tone (coin sound). If subjects failed to reach the correct target, the trial was aborted and a demotivating low-pitched tone was played. Failure trials included aborted trials due to ocular fixation breaks, incorrect reaches to locations on the screen outside the tolerance window (3 cm radius) around the valid target(s), and reaches later than the maximal response time. Subjects were explicitly requested to respond as accurately and as rapidly as possible.

Subjects had to perform the rule-selection task in two variants, which differed only in the instructed trials, not the choice trials. The idea was to induce a graded level of predictability (PROB task) or preferability (AMNT task) between the two possible rules. For this, the relative size of the two triangles in the pre-cue was varied in seven steps corresponding to seven instructed expectation levels. Note, for convenience we refer to these levels jointly as “bias conditions” even though the behavioral bias that will be potentially induced by this task parameter is our tested variable. In the zero-bias trials the triangles had equal size. In the 100% bias level conditions either only the *cw* or the *ccw* triangle was visible and larger than in the zero-bias condition. In the intermediate bias conditions the two triangles had intermediate sizes. The seven instructed-bias conditions used the following combinations of pre-cue triangles (base lengths of the triangle): {3.5:0.0, 3.0:0.7, 2.5:1.4, 2.0:2.0, 1.4:2.5, 0.7:3.0, 0.0:3.5}. The bias level was kept constant within each block of six trials as will be described below.

### Probabilistic rule-selection task (PROB)

The idea of the PROB task was to induce a graded level of rule predictability across trials, without a difference in the final value of the two motor-goal options. In the PROB task, we assigned the colors magenta and cyan to the two pre-cue triangles. The size of the pre-cue triangles indicated the likelihood with which the *cw* and *ccw* rules would be instructed by the rule-cue later in the trial. The reward for the targets associated with either rule was identical. Seven bias levels corresponded to likelihoods of {6:0, 3:1, 2:1, 1:1, 1:2, 1:3, 0:6} for an instruction of the *ccw* or *cw* rule, respectively. In the case of a bias level 2:1 toward *ccw*, subjects were offered 4 *ccw* trials and 2 *cw* trials. Subjects received three reward units for each correctly performed instructed trial and 1.5 reward units for either choice in the choice trials. Thus, at the 100% bias levels (6:0 or 0:6), the low probability rule had 0% chance of getting 3 units in instructed trials and 100% of getting 1.5 units in choice trials, resulting in an expected value of 0 × 3 × 2/3+1 × 1.5 × 1/3 = 0.5 reward units. The high probability rule had a 100% chance of getting 3 units in instructed trials and 100% chance of getting 1.5 units in choice trials, resulting in an expected value of 1 × 3 × 2/3+1 × 1.5 × 1/3 = 2.5 reward units. The ratios of initially expected values (EV) associated with the two rules at the seven bias levels were then {2.5:0.5, 2:1, 1.83:1.17, 1.5:1.5, 1.17:1.83, 1:2, 0.5:2.5}. Note that these initial EVs were only valid for the time between the pre-cue and the rule-cue. After the rule-cue, the final value for instructed trials was 3 for the instructed rule, zero for the non-instructed rule, and 1.5 for both rules in the choice trials.

### Reward-amount based rule-selection task (AMNT)

The idea of the AMNT task was to induce a graded preference for the different options without being encouraged to plan an according movement. In the AMNT task we assigned colors orange and green to the two pre-cue triangles. The size of the pre-cue triangles indicated the amount of reward that would be associated with each rule in case it was instructed later in the trial. The probability of each rule being instructed was kept 50:50. The reward units at the seven bias levels corresponded to {6:0, 5:1, 4:2, 3:3, 2:4, 1:5, 0:6}. For example, in the case of a bias level 4:2 toward *ccw*, subjects got 4 reward units for an instructed *ccw* reach and 2 units for an instructed *cw* reach, whereas in the case of a bias level 6:0 toward *cw*, subjects received six reward units for *cw* reach but nothing for *ccw* reach (but still have to reach to the unrewarded target to complete that experimental block and proceed to the next block). The length of the feedback sound at the end of successful trials matched the amount of reward subjects received in that given trial. The ratios of initial EV associated with the two rules at the seven bias levels were then {2.5:0.5, 2.17:0.83, 1.83:1.17, 1.5:1.5, 1.17:1.83, 0.83:2.17, 0.5:2.5}. Again, these EVs were only valid for the time between pre-cue and rule-cue. After the rule-cue, the final EV for instructed trials was equal to the reward units assigned to the instructed rule (6:0, 5:1, etc.), always zero for the non-instructed rule, and 1.5 for both rules in the choice trials.

Importantly, we matched the preliminary EVs in the PROB and AMNT tasks as closely as possible, given the block structure of trials. In five out of seven conditions the EVs matched exactly. In two conditions the EV ratios matched approximately (PROB 2:1, AMNT 2.17:0.83). Choice trials and zero-bias trials were identical between both tasks in all other respects. Thus, prior to the rule-cue (when subjects did not know yet if a given trial will be instructed or choice) and after the rule-cue, the EVs for choice trials in both tasks matched. Hence our task design ensures that any observed differences in the free-choice behavior between the two tasks should be attributable to biases that were introduced by the purposeful manipulation of the expectation for the instructed trials, and not to differences in the choice trials.

We also ran a control experiment for the AMNT task in which we doubled the reward contrast between high- and low-valued options. The reward units at the seven bias levels in this AMNT-double task corresponded to {12:0, 8.5:0.5, 5:1, 3:3, 1:5, 0.5:8.5, 0:6}. Sixteen subjects who had previously participated to the AMNT experiment were invited to perform the AMNT-double task. The subgroup selection depended only on subject availability and was independent of previous performance on the AMNT task.

### Subject compensation and bonus

Recording sessions terminated when subjects reached 600 successful trials. By design, the same amount of reward was reached in both types of task. In the PROB task, in one block of six trials, the four instructed trials (4 × 3 units) and two choice trials (2 × 1.5 units) led to 15 reward units. To reach 600 trials, subjects needed to complete 100 blocks, i.e., a total of 15 × 100 = 1500 units. In the AMNT task, in each biased block, two out of four instructed trials delivered high reward and another two delivered low reward, e.g., in a 1:5 condition block, subjects received (5 × 2) + (1 × 2) = 12 reward units. Two choice trials (2 × 1.5 units) added to the same total of 15 units per block as in the PROB task. We converted three reward units to 2 Euro cent, which finally made 2/3 × 1500 units = 1000 cent, thus €10 per session. As there was no penalty for aborted trials and subjects had to reach the same number of successful trials, the total compensation per session was identical between tasks.

Additional to the baseline compensation of €10 for each accomplished session, a bonus of up to €6 for good performance could be achieved: performance under 50%: no bonus; 50%: bonus of €1; then each step of 5% will add €0.5 until reaching maximal bonus of €6 at 100%). Alternatively, subjects received a compensation of €6 per hour, if this yielded the higher compensation. For example, subjects with very high performance typically spent about 1 h and received €15–16 whereas subjects who made many error trials and/or multiple pauses (self-paced task design) spent about 2 h in the setup and received €12–13).

### Pre-recording procedure and balancing

Subjects were required to maintain gaze at the center of the screen. For this, a calibration of the eye-tracking system was first carried out. Then a short (5–10 min) training session was run to accustom subjects to the task and setup condition. Since our experiment aimed at quantifying biasing effects, we wanted subjects to explore the range of possible free-choice responses before the start of the experiment. For this, we ran an initial balancing session for each subject to discourage subjects from repeating the same default reach choices through the rest of the experimental session. The balancing task contained only trials with a zero-bias condition (equal triangle sizes) and differed from the rule-selection task described above only in the reward schedule that we applied on the choice trials. Instead of rewarding both options with 100% probability, we used a bias-minimizing reward schedule (BMRS). In the BMRS the reward probabilities for free-choice targets were calculated based on the individual subject's choice history. The less often a target was freely chosen in the previous two choice trials, the higher the reward probability in favor of this target was (Klaes et al., 2011):

$$\begin{array}{l} p\left(R_{cw}\right)=F\left(n_{ccw}-n_{cw}\right)\\ p\left(R_{ccw}\right)=F\left(n_{cw}-n_{ccw}\right) \end{array}$$

where *n*_*cw*_ is the total number of rewarded *cw* reaches and *n*_*ccw*_ is the total number of rewarded *ccw* reaches. *F* was defined as:

$$F\left(x\right)=\{\begin{array}{cc} \;1, & \text{x }>1\\ 2/3, & \text{x }=1\\ 1/2, & \text{x}=\text{0 }.\\ 1/3, & \text{x}=-\text{1}\\ \;0, & \text{x }<\;-\text{1} \end{array}$$

Subjects were explicitly told that chosen targets would successively stop being rewarded and they needed to explore all possible reaches to complete this task. The balancing task was run until the subject made at least two *cw* and two *ccw* reaches at each pre-cue position, which means at least 16 choice trials. As choice trials made up 33% of all trials, the balancing task then comprised a minimum of 48 trials.

### Apparatus and data acquisition

Subjects were seated in a dimly lit room facing an LCD screen (19” ViewSonic VX922) mounted behind a transparent touch sensitive screen (IntelliTouch, ELO Systems, CA, USA), with a chinrest and forehead band used to stabilize head position. The screen was mounted with a tilt of 33° from the vertical for subject's comfort, with the lower edge on the table at ~40 cm distance from the chinrest base and the top edge at eye level. The luminance of all colored stimuli was in the range 12–13 cd/m^2^ (luminance meter LS-100, Minolta, Japan). Luminance was measured at eye level when positioning the color cues at the top of the four positions used in the experiment, i.e., at a direction of 90° from the screen center and with an eccentricity of 9 cm. Throughout the trial, the gaze direction of the subjects was constrained at the central fixation point (red square) within a tolerance window of 3 cm (~4.3° VA radius). Eye positions were monitored by a camera placed in front of the screen's lower edge (Eyelink 1000, Kanata, Canada). A real-time LabView program running on a PXI computer (National Instruments) was used to control the tasks and to register relevant stimulus properties, event timings, and subject's behavioral responses in each trial.

### Behavioral data analysis

The main goal of this study was to quantify the biasing effect of predictability and preferability on choice behavior. Since preliminary analysis revealed symmetric effects of *cw/ccw* rule in our data (effects of interaction between bias degrees and rule types (*cw*-*ccw*) on reaction times in instructed *follow* trials: PROB task: t-statistic = −1.59, *p* > 0.05; AMNT task: t-statistic = 0.11, *p* > 0.05; interaction between bias degrees and rule types (*cw*-*ccw*) on choice probabilities: PROB task: t-statistic = −0.07, *p* > 0.05; AMNT task: t-statistic = −0.44, *p* > 0.05, GLMM; see details on GLMM below), we chose the absolute value of the pre-cued bias level as the independent variable and merged all trials with different pre-cue positions. In other words, we grouped the data into four bias conditions, one zero-bias condition plus three non-zero bias conditions. Bias degrees were quantified by the contrast in preliminary EV associated with each pair of reward amount or probability: higher EV−lower EV/higher EV + lower EV. Bias degrees were {0, 0.22, 0.33, 0.67} for the PROB task and {0, 0.22, 0.45, 0.67} for the AMNT task.

Additionally, we sorted the data according to rule-congruency, i.e., according to whether the reach was conducted to the same (*follow*) or the opposite (*against*) direction as the direction indicated by the bigger pre-cue triangle (bias direction). Note that *follow* and *against* responses could occur by instruction in instructed trials and by subjects' choice in choice trials and the instructed trials of the probabilistic task are equivalent to typical cue-validity tasks in which *follow* trials would correspond to *valid* trials, and *against* trials to *invalid* trials.

We analyzed error rate (ER), reaction time (RT) in both error and correct trials, and movement time (MT) in instructed trials. ERs were defined as the fraction of trials not leading to a successful target acquisition within the reach period, either due to miss-reaching or fixation breaks (often occurring together). As errors other than miss-reaches were in general very rare and both targets were considered valid in choice trials, we report ERs and error RTs only in instructed trials. RTs were defined as the time between the go-signal and the subject's release of the touch screen from the fixation position and MTs as the time between the subject's release of the fixation point to the time that the subject's finger arrived at the target position. Both RTs and MTs were corrected for display and touch screen delays. Trials with invalid RTs (0.5% of total number of trials) were excluded from the RT analysis as subjects might have prematurely released the screen before the rule-cue was perceived. As rejection threshold we used 2.5 interquartile ranges below Q1 (25% quartile) or 100 ms, whichever value was higher.

We analyzed RTs and choice probability (CP) in choice trials. CP was defined as the fraction of correct choices following the bias introduced by the pre-cue. For the zero-bias condition we show the fraction of *cw* choices.

We tested for biasing effects in all aforementioned dependent variables. For this, a generalized linear mixed model (“fitglme”; MATLAB R2014b) was fitted to assess influences of bias degrees on ER, RT, MT, and CP, as well as differential effects between PROB and AMNT tasks. Full models included the factors bias degree (Bias: continuous variable), rule congruency (Congruency: categorical responses *follow* vs. *against* biased direction), and task type (Task: categorical variable: AMNT vs. PROB) and all interaction terms, as fixed effects. Note that we considered ER, RT, and MT at zero-bias degree in both *follow* and *against* categories to keep the zero-bias level included in both *follow* and *against* fittings. Subjects were included as random effect (uncorrelated random intercepts and slopes for bias levels, congruency, and tasks) to account for the variance across subjects. The likelihood of the models including or excluding different fixed and random effects were compared using the Matlab function “compare(model1, model2).”

We used the following model to test overall differential effects of bias between *follow*-*against* responses on ER (binomial response), RTs, and MTs with interaction term between Bias and Congruency in each task separately:

(M1) $$X\;\sim \;Bias{\;}^{*}\;Congruency\;+\;(Bias{\;}^{*}\;Congruency\;|\;Subjects),$$

and to test the differential effects on ERs, RTs, and MTs between tasks:

(M2) $$\begin{array}{l} X\;\sim \;Bias{\;}^{*}\;Congruency{\;}^{*}\;Tasks\\ \;+\;(Bias{\;}^{*}\;Congruency{\;}^{*}\;Tasks\;|\;Subjects). \end{array}$$

Next, only for RTs, we additionally tested the differential effects on error RTs between tasks using the model:

(M3) $$\begin{array}{l} RT\;\sim \;Bias{\;}^{*}\;Error\;(success/error){\;}^{*}\;Tasks\\ \;+\;(Bias{\;}^{*}\;Error{\;}^{*}\;Tasks\;|\;Subjects). \end{array}$$

When instructed to go against the bias, DDMs predict short error RTs in case of a bias mechanism mediated by a baseline shift whereas long error RTs in case of a drift rate change (Simen et al., 2009; Leite and Ratcliff, 2011). As errors were rare in instructed *follow* trials and choice trials, we inspected error RTs only in instructed *against* trials.

**Figure 2:**
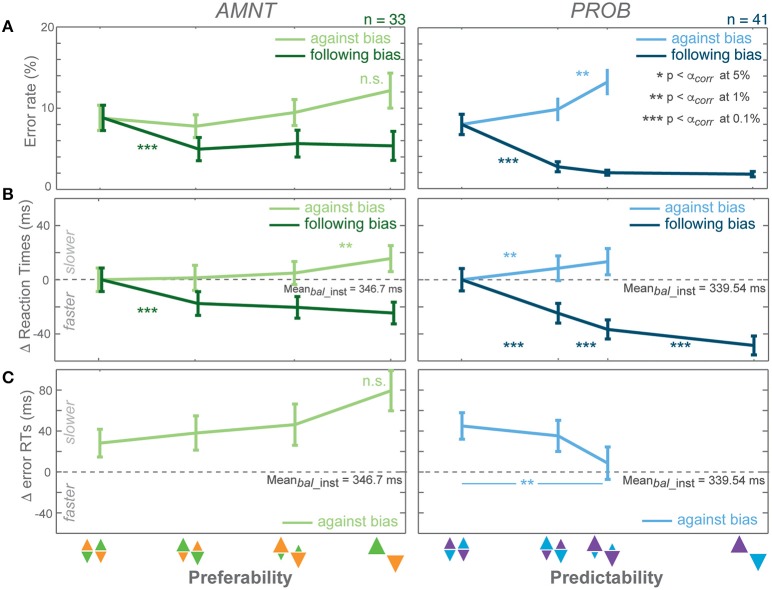
**Biasing effects on instructed responses**. **(A)** Average error rates (ER): proportion of error trials committed during the reach period, **(B)** average reaction time (RT) difference from average RTs in instructed trials of zero-bias condition of each task in all bias levels in AMNT (left) and PROB (right) tasks, **(C)** average error RT difference (zero line indicates average correct RTs in instructed trials of zero-bias condition of each task). Dark and light colors represent *follow* and *against* responses, respectively. Error bars depict standard errors. (^*^ *p* < α_*corr*_ at 5%, ^**^ *p* < α_*corr*_ at 1%, ^***^ *p* < α_*corr*_ at 0.1%, paired *t*-test with *Bonferroni* correction).

When there was a biasing effect, we asked further whether (a) the biasing effect was symmetric for costs (*against*) and benefits (*follow*) and whether (b) the biasing effect was graded, i.e., scaled with the strength of the bias signal. With the model M1 we computed the slopes for *follow* and *against* and compared their confidence intervals to test whether the absolute values of the slope differed (asymmetry) or overlapped (symmetry) between both conditions. Note that the obviously different slopes (Figure 2B) between *follow* and *against* conditions were the reasons why we introduced congruency as a factor in the model. By modeling the data separately for each “branch” of the bias factor separately, we got better linear fits than when treating the seven bias levels as a single factor (data not shown). We tested for graded biasing effects vs. a single step-like effect of bias, with *post-hoc* tests (paired *t*-tests with *Bonferroni* corrections for multiple comparisons) comparing each pair of successive bias conditions.

We tested biasing effects on CP using a separate full model without the *Congruency* term as there was no *follow*-*against* distinction in this case:

(M4) $$\begin{array}{l} CP\;(binomial\;response)\;\sim \;Bias{\;}^{*}\;Tasks\\ \;+\;(Bias{\;}^{*}\;Tasks\;|\;Subjects) \end{array}$$

With additional *post-hoc* tests corrected for multiple comparisons we tested the graded effect as introduced above.

## Results

We probed the biasing effect of motor-goal preferability and predictability on later risk-free choices in two steps. First, we wanted to confirm that both our value and probability manipulations were effective in affecting subject behavior. To do so, we analyzed error rates (ER), reaction times (RT), and movement times (MT) in instructed trials. If the pre-cue had a biasing effect then there should be costs and benefits involved with having to go *against* or being allowed to *follow* one's internal bias as a result of an instruction. Specifically, *follow* trials should lead to faster RTs and lower ER than *against* trials. Second, we wanted to test if preferability and predictability have similar or differential biasing effects on later choice behavior. For this, we analyzed RTs and choice probabilities (CP) in choice trials. If preferability and predictability induce the same biasing mechanism, we should observe the same response pattern across tasks. If instead preferability and predictability induce different biasing mechanisms, we should observe different response patterns. The former hypothesis might still be supported if the behavioral response patterns in instructed and choice trials are the same in the PROB and AMNT task, except for a potentially reduced effect in the AMNT task compared to the PROB task (Maddox and Bohil, 1998; Mulder et al., 2012).

### Performance comparison PROB vs. AMNT task

We first tested if the AMNT and the PROB experiment were different in task difficulty. For this we did not compare only the total performance, but particularly the zero-bias trials. Zero-bias trials were identical in both experiments, except for the task context.

Average performance was 86.84 ± 2.1% in AMNT task (*N* = 33) and 91.53 ± 0.8% (mean ± SEM) in PROB task (*N* = 41). Subjects who participated in both tasks showed slightly better overall performance in PROB task than AMNT task (AMNT: 88.13 ± 1.7%, PROB: 91.91 ± 0.8%, mean ± SEM, *N* = 31, *p* < 0.05, paired *t*-test). However, importantly, when comparing performance only in the zero-bias condition of the instructed trials, which served as our reference condition, subjects performed equally well (AMNT: 87.53 ± 1.75%, PROB: 89.67 ± 1.37%, mean ± SEM, *N* = 31, *p* > 0.05, paired *t*-test).

Subjects performed both tasks with shorter RT in instructed compared to choice trials (AMNT [instructed-choice]: −24.48±2.47 ms, *p* < 10^−9^, PROB [instructed-choice]: −38.00±4.24 ms, *p* < 10^−9^, mean ± SEM, *N* = 31, paired *t*-test) and overall responded faster in PROB task than in AMNT task (AMNT-PROB: 21.03 ± 6.62 ms, *p* < 0.01, mean ± SEM, *N* = 31, paired *t*-test). However, when comparing only RTs from the zero-bias condition, subjects showed no RT difference between tasks (inst [AMNT-PROB]: 7.95 ± 7.87 ms, *p* > 0.05, choice [AMNT-PROB]: −0.31±8.07 ms, *p* > 0.05, mean ± SEM, *N* = 31, paired *t*-test).

In summary, the AMNT and the PROB task contexts did not lead to performance differences in the zero-bias trials, i.e., in the trials which do not differ between the tasks. In trials with non-zero bias levels, subjects responded on average more quickly and made fewer errors in the PROB task than in the AMNT task.

### Effects of *a priori* preferability vs. predictability in instructed behavior

We first needed to establish whether our manipulations of probability and amount were strong enough to be effective. We compared ERs, RTs, and MTs in instructed trials between the PROB and AMNT task to test if they depended on the bias degree. If so, we further tested if bias degree led to symmetry in costs and benefits, and if the effect was gradually increased with increasing bias degree. In both tasks we found significant effects of bias degree on instructed behavior, but with differences in individual aspects, as detailed in the following.

#### Error rates (ER)

Error rates (ER) depended on the instruction to follow or go against the bias in both tasks (Figure 2A). A generalized linear mixed effect model (GLMM) showed higher ERs when subjects were instructed to reach against the bias than follow the bias in both tasks (interaction between *Bias* and *Congruency*: AMNT: t-statistic = 6.07, *p* < 10 ^−8^; PROB: t-statistic = 10.05, *p* < 10^−9^, *N* = 43). The effect on ERs differed between the PROB and AMNT task quantitatively. The ER full model confirmed that the ER cost was significantly higher in *against*-instructed trials in the PROB task than in the AMNT task (interaction between *Bias* and *Tasks* on *against* trials: t-statistic = −3.47, *p* < 0.001). Further there were overall significant difference in ER patterns between tasks (interaction between *Bias, Congruency*, and *Tasks*: t-statistic = −3.96, *p* < 10^−4^).

The ER dependency on the bias degree was symmetric in strength in instructed trials of both tasks. Decrease in response to *follow* instructions was not significantly steeper than the ER increase in response to *against* instructions. (AMNT-*against*: 95% confidence interval CI = [−0.01, 1.03] (increase in percentage of error trials per one bias degree); AMNT-*follow*: CI = [−2.89, −0.97]; PROB-*against*: CI = [1.41, 3.21]; PROB-*follow*: CI = [−3.61, −1.92]).

ER in neither tasks showed a gradual effect as function of bias degree (Figure 2A).

#### Reaction times (RT)

Reaction times (RT) depended on the bias degree in both tasks similarly to ERs, but partially in a more gradual fashion (Figure 2B). GLMM showed an overall differential effect of biasing degree on RT between *follow* and *against* instructed reaches in both tasks (interaction between *Bias* and *Congruency*: AMNT: t-statistic = 9.72, *p* < 10^−9^; PROB: t-statistic = 12.28, *p* < 10^−9^). This means the bias degree induced systematic costs and benefits for RTs in instructed reaches. Also, the RT full model showed significantly different patterns of RTs between tasks (interaction between *Bias, Congruency*, and *Tasks*: t-statistic = 6.23, *p* < 10^−9^), and a smaller RT benefit in the AMNT task as compared to the PROB task (interaction between *Bias* and *Tasks*: t-statistic = −5.65, *p* < 10^−9^).

RT costs and benefits in the instructed trials of each task were not symmetric. The PROB task showed a significantly larger RT benefit of following than cost for going against the bias (PROB-*against*: CI = [16.99, 46.79] (increase in RTs (ms) per one bias degree); PROB-*follow*: CI = [−85.31, −63.71]). Asymmetry was not as strong in the AMNT task, since the absolute values of the confidence limits partly overlapped (AMNT-*against*: CI = [12.94, 27.50]; AMNT-*follow*: CI = [−44.41, −27.39]).

RTs in instructed trials partially showed a gradual increase in effect strength with increasing bias degree (Figure 2B). In the AMNT task, RTs decreased in one significant step when subjects were instructed to follow the bias. In the PROB task RTs decreased with each step in response to *follow* instructions with increasing bias level.

#### Analysis of error RTs

Depending on which mechanism explains the decision process, RTs in unsuccessful trials can be expected to be either faster or slower than successful trials (e.g., Smith and Ratcliff, 2004; Bogacz, 2007; Brown and Heathcote, 2008; Heitz, 2014). We analyzed error RTs when an instruction to go against a bias was violated; since error rates in *follow* trials were too rare to be analyzed properly. Error RTs depended on the bias condition in opposite ways in the AMNT and PROB task. Error RTs showed significant differential effects in response to *follow* or *against* instructions in the PROB task, but not in the AMNT task (interaction between *Bias* and *Congruency*: AMNT: t-statistic = −1.07, *p* > 0.05; PROB: t-statistic = 2.59, *p* < 0.01). Due to limited number of errors in *follow* trials of both tasks, slope analyses detected no significant slopes in *follow* trials (AMNT-*follow*: CI = [−36.90, 67.87]; PROB-*follow*: CI = [−64.10, 7.51]; not shown). Importantly, in *against* trials, slope analyses revealed significant increase in error RTs with bias levels in AMNT task, as opposed to significant decrease in PROB task (AMNT-*against*: CI = [8.64, 80.66]; PROB-*against*: CI = [−127.78, −50.155]; Figure 2C). RT differences between individual consecutive bias levels did not reach significance in either task, likely due to the limited number of error trials.

Overall, analysis of the instructed trials showed that manipulation of both preferability and predictability were effective and had consequences on RTs and ERs, yet, with indications from the error trials analysis that underlying mechanisms might differ.

### Effects of *a priori* preferability vs. predictability on free-choice behavior

The main goal of our study was to investigate the effects of prior probability and expected amount on risk-free, reward-balanced choice behavior. We quantified choice probabilities (CP), and RTs for the randomly interspersed choice trials.

#### Choice probabilities (CP)

**Figure 3:**
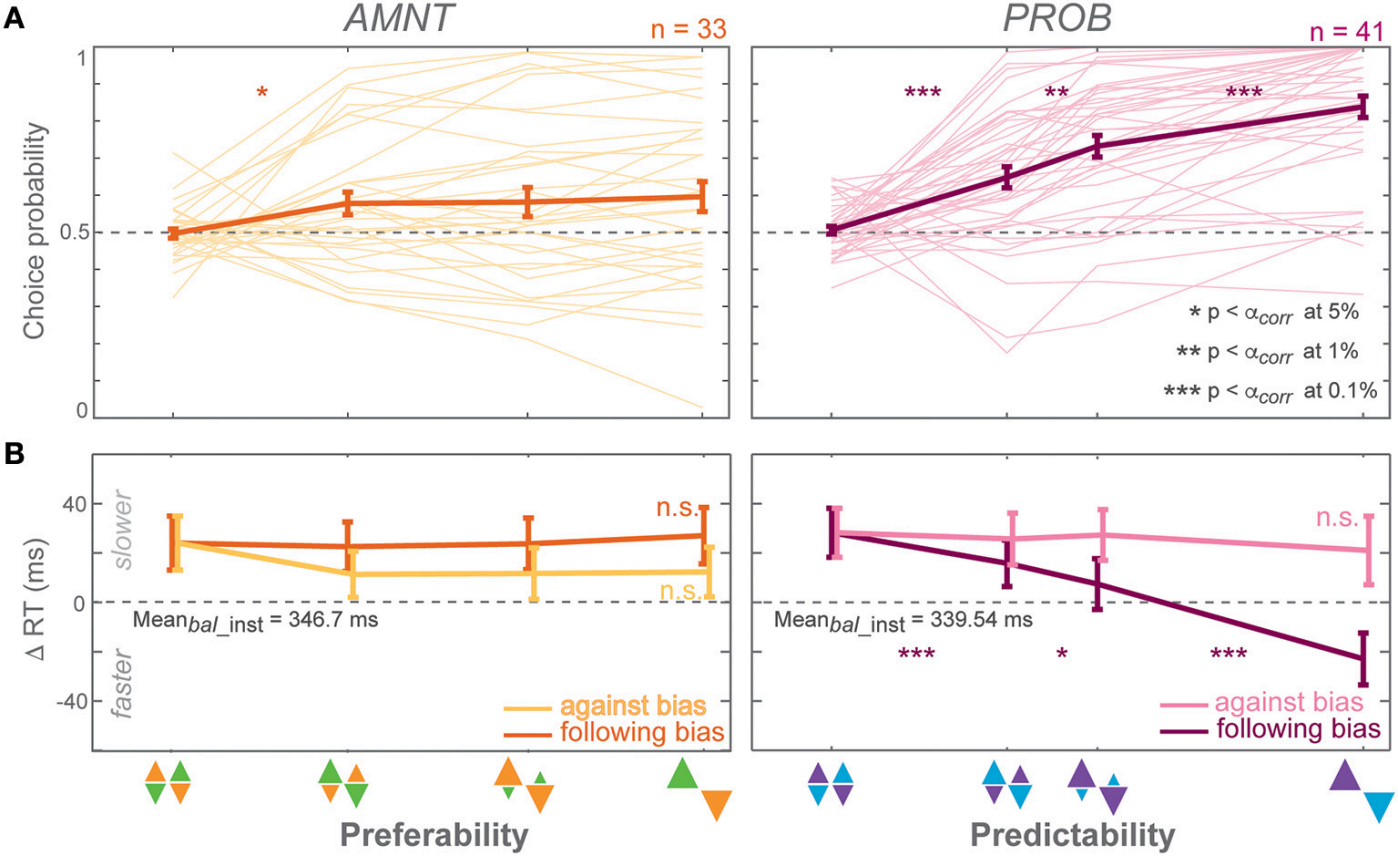
**Biasing effects on free-choice responses**. **(A)** Average choice probability (CP): fractions of choice toward the biased direction (at the zero-bias level, as there is no biased direction, fractions of clockwise choice are presented), and **(B)** average reaction time (RT) difference (zero line indicates average RTs in instructed trials of the zero-bias condition of each task) in all bias levels in AMNT (left) and PROB (right) tasks. Dark and light colors represent *follow* and *against* responses, respectively. The error bars represent standard errors. (^*^ *p* < α_*corr*_ at 5%, ^**^ *p* < α_*corr*_ at 1%, ^***^ *p* < α_*corr*_ at 0.1%, paired *t*-test with *Bonferroni* correction).

Choice probabilities (CP) depended on the bias degree in both tasks (AMNT: t-statistic = 2.14, *p* < 0.05; PROB: t-statistic = 7.90, *p* < 10^−9^), however with a significantly smaller biasing effect in AMNT task than in PROB task (interaction between *Bias* and *Tasks*: t-statistic = −6.88, ^*^p < 10−11). *Bonferroni* corrected *post-hoc t*-tests revealed a graded biasing effect in the PROB task, in which choice bias became stronger for each step of bias degrees. In contrast, choice bias in the AMNT task showed only a single significant step between balanced and [1/3] bias degree, and no difference between different non-zero bias degrees (Figure 3A).

#### Choice reaction times (RT)

Choice reaction times (RT) were only affected by bias degree in the PROB task. GLMM showed a differential effect between *follow*-*against* reaches in PROB task but not in the AMNT task (interaction between *Bias* and *Congruency*: AMNT: t-statistic = −1.91, *p* > 0.05; PROB: t-statistic = 7.02, *p* < 10^−9^). The RT full model confirmed significantly different patterns of RTs between tasks (interaction between *Bias, Congruency*, and *Tasks*: t-statistic = 7.16, *p* < 10^−9^) with the PROB task showing a significantly larger effect on RT benefits when following the bias (interaction between *Bias* and *Tasks*: t-statistic = 8.49, *p* < 10^−9^). In addition, a clear asymmetry of choice RTs was revealed in the PROB task (PROB-*against*: CI = [−30.16, −4.44]; PROB-*follow*: CI = [−92.78, −61.25]).

RTs in the choice trials showed a gradual increase in effect strength with increasing bias degree only for benefits in the PROB task. *Post-hoc* tests showed no significant RT differences between individual neighboring bias degrees in the AMNT task but RTs that increased gradually with the bias degree in *follow*-choices in the PROB task (Figure 3B).

Notably, the RT benefit in *follow* reaches was comparable between instructed and choice trials in the PROB task, while in the AMNT task there was only a benefit for *follow* instructions, not for *follow* choices (interaction between *Bias* and trial types: AMNT: t-statistic = −1.21, *p* < 10^−9^; PROB: t-statistic = 7.02, *p* > 0.05). This means, while predictability affected later choice behavior in the same way as instructed behavior, in contrast, preferability showed clear effects in instructed behavior but did not generalize to the choice behavior.

### No effect of doubling the reward in the AMNT task

The observed limited effects of preferability as compared to predictability might be due to lack of EV contrast. EVs cannot be linearly translated into subjective expected utility which is more directly linked to choice (Von Neumann and Morgenstern, 1944; Savage, 1954). To rule out this possibility, we conducted a control experiment in which we doubled the reward amount ratio between high- and low-value options.

#### Error rates (ER)

**Figure 4:**
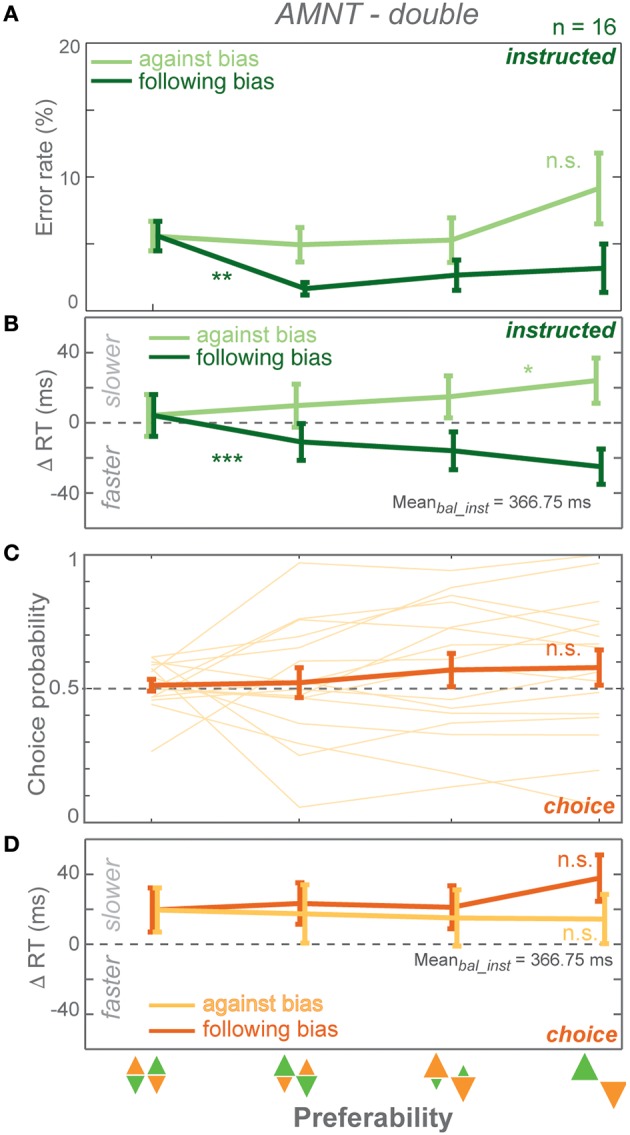
**Results of AMNT task with double reward size (AMNT-double)**. **(A)** Average error rates, **(B)** average instructed RT difference, **(C)** Choice probabilities, **(D)** average choice RT difference (zero line indicates average RTs in instructed trials of zero-bias condition of each task) in all bias levels. Dark and light colors represent *follow* and *against* responses, respectively. Error bars represent standard errors. (^*^ *p* < α_*corr*_ at 5%, ^**^ *p* < α_*corr*_ at 1%, ^***^ *p* < α_*corr*_ at 0.1%).

Error rates (ER) depended on the instruction to follow or go against the bias in the AMNT-double task. A GLMM showed higher ERs when subjects were instructed to reach against the bias than follow the bias (interaction between *Bias* and *Congruency*: t-statistic = 3.86, *p* = 0.0001; *N* = 16). ER decrease in response to *follow* instructions was significantly steeper than the insignificant increase in response to *against* instructions. (*against*: CI = [−0.40, 1.29]; *follow*: CI = [−5.00, −1.14]). Similarly to the standard AMNT task, ERs decreased when subjects were instructed to follow the bias (Figure 4A).

#### Reaction times (RT)

Reaction times (RT) depended on the bias degree in the AMNT-double task similarly to the standard AMNT task. GLMM showed an overall differential effect of biasing degree on RT between *follow* and *against* instructed reaches in both tasks (interaction between *Bias* and *Congruency*: t-statistic = 7.10, *p* < 10^−9^). As in the standard AMNT task, asymmetry was weak or absent in the AMNT-double task, since the absolute values of the confidence limits partly overlapped (*against*: CI = [14.43, 39.95]; *follow*: CI = [−60.03, −30.71]). RT decrease in *follow* trials and increase in *against* trials showed similar pattern as in the standard AMNT task (Figure 4B).

#### Choice probabilities (CP)

Choice probabilities (CP) showed insignificant increase with bias degrees in the AMNT-double task (t-statistic = 1.62, *p* > 0.05), and no CP difference was detected between the standard AMNT and the AMNT-double tasks (interaction between *Bias* and *Tasks* (AMNT vs. AMNT-double): t-statistic = 0.1114, *p* > 0.05).

#### Choice RTs

Choice RTs were not affected by bias degree in the AMNT-double task. GLMM showed no differential effect between *follow*-*against* reaches in the AMNT-double task (interaction between *Bias* and *Congruency*: t-statistic = −1.91, *p* > 0.05).

CPs and choice RTs showed no difference between neighboring bias degrees (Figures 4C,D).

In summary, doubling of the reward amount contrast between high- and low-value options did not change the strength of biasing effects in the AMNT task.

### Subject sub-grouping based on choice bias

CPs showed a weaker bias in the AMNT task than in the PROB task on average across all subjects. We asked if this was because some subjects showed no bias in the AMNT task, while others might show a bias of the same magnitude as in the PROB task. If so, would the pattern of RT results for subjects with a strong CP bias in the AMNT task look similar to the pattern of RT results in the PROB task? CPs across subjects varied, especially in the AMNT task. We subdivided subjects depending on their bias in the choice probabilities to test, first, if individual subjects showed a choice pattern contrary to the average pattern described above; and second, whether subjects' choice behavior in choice trials would predict the RT patterns in instructed and in choice trials. Using a GLMM on each subject's CP, we distinguished three classes of subjects: CP-biased (significant positive slope), CP-unbiased (slope not significantly different from zero), or CP-counter-biased (significant negative slope).

Most subjects in PROB task were CP-biased (33 biased, 8 unbiased, and no counter-biased). In contrast, the majority of subjects in the AMNT task were CP-unbiased (10 biased, 20 unbiased, and 3 counter-biased). Out of the 31 subjects who participated in both experiments, 27 were biased and 4 were unbiased in the PROB task. Out of these 27 biased subjects in the PROB task, 9 were biased, 17 were unbiased, and one was counter-biased in the AMNT task. And out of four unbiased subjects in the PROB task, two were also unbiased and two were counter-biased in AMNT task. No subject showed CP-bias in the AMNT task and was unbiased or counter-biased in the PROB task. In summary, no individual subject showed a reversed pattern of CPs in the AMNT task to the average pattern of CPs.

In the PROB task, RT benefits of instructed *follow* reaches matched RT benefits when choosing *follow* reaches in choice trials in both biased and unbiased subjects. GLMM showed no interaction between *Bias* and trial types (instructed vs. choice) in either subgroup [PROB (biased): t-statistic = 0.82, *p* > 0.05; PROB (unbiased): t-statistic = 1.49, *p* > 0.05]. The biasing effect differed between subject subgroups on instructed RTs but not in choice RTs [interaction between *Bias* and subgroup: PROB (inst): t-statistic = 2.83, *p* < 0.01; PROB (choice): t-statistic = 1.06, *p* > 0.05]. Notably, shorter RTs when choosing *against* bias seemed to come from unbiased subjects while biased subjects showed flat RTs in *against* trials of both tasks [AMNT-*against* (biased): t-statistic = −1.96, *p* > 0.05; AMNT-*against* (unbiased): t-statistic = −2.78, *p* < 0.01; PROB-*against* (biased): t-statistic = −1.07, *p* > 0.05; PROB-*against* (unbiased): t-statistic = −2.86, *p* < 0.01].

**Figure 5:**
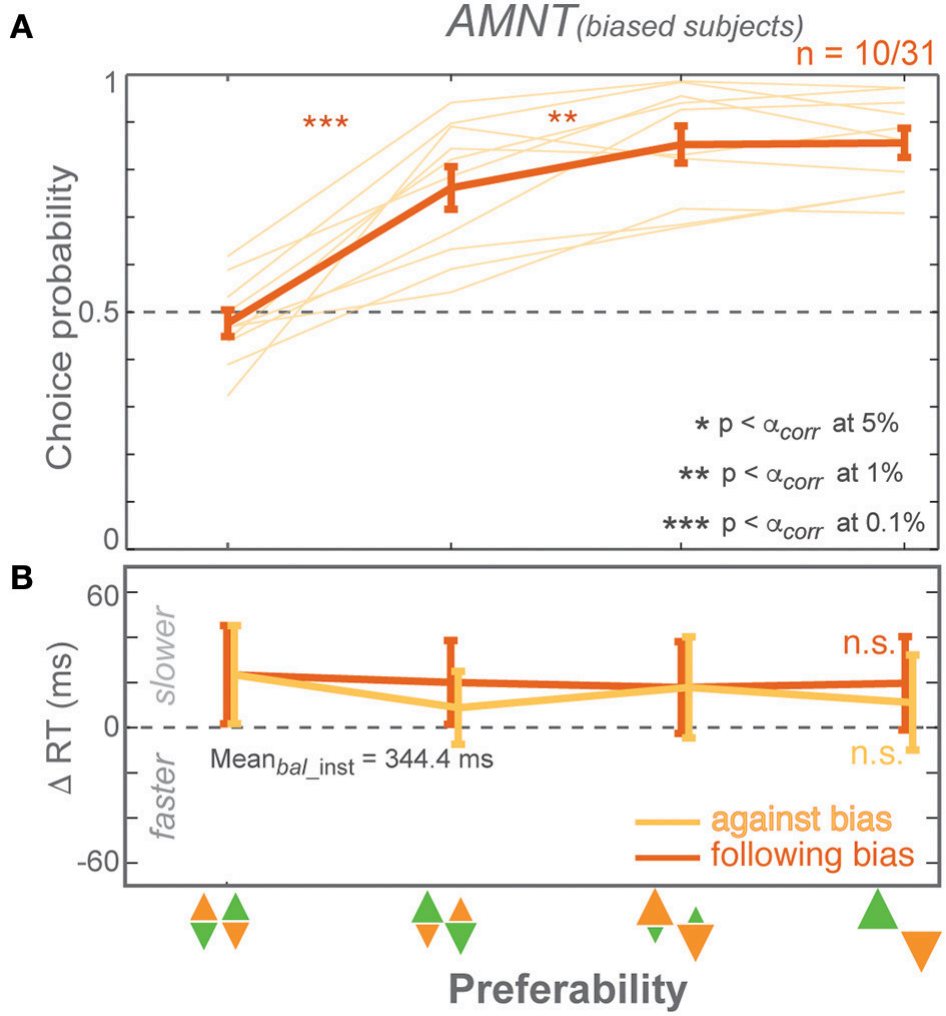
**Responses of CP-biased subjects in AMNT task**. **(A)** Choice probabilities, and **(B)** average choice RT difference (zero line indicates average RTs in instructed trials of zero-bias condition) in all bias levels. Dark and light colors represent *follow* and *against* responses, respectively. The error bars depict standard errors. (^*^ *p* < α_*corr*_ at 5%, ^**^ *p* < α_*corr*_ at 1%, ^***^ *p* < α_*corr*_ at 0.1%).

In summary, dividing subjects into CP-biased and CP-unbiased subgroups showed that even the minority of subjects who were biased in their choice behavior in the AMNT task lack a biasing effect on choice RTs (Figure 5). This also means they showed significantly different RT behavior between the AMNT and PROB tasks and further supports the idea that the underlying decision processes are different in both tasks.

### Movement times

Studies on motor planning had previously shown that invalid pre-cueing can affect not only RT but also MT (e.g., Leis et al., 2005). While classical DDMs to not consider MT, in the context of affordance or motor-oriented models of decision-making, motor kinematics can reveal additional insights (Gallivan et al., 2015). The prediction would be that having to go against planned movement should require any preliminary motor plan to be suppressed and lead to slower movement execution (Cisek, 2012).

#### Movement time (MT) analysis

GLMM showed a differential effect on MT between *follow* and *against* reaches in both tasks, yet the effect in the AMNT task was minimal (interaction between *Bias* and *Congruency*: AMNT: t-statistic = 2.01, *p* < 0.05; PROB: t-statistic = 5.76, *p* < 10^−8^). Correspondingly, the MT full model confirmed significantly different patterns of MTs between tasks (interaction between *Bias, Congruency*, and *Tasks*: t-statistic = −5.03, *p* < 10^−6^), and a substantially higher MT of *against* reaches in the PROB task as compared to the AMNT task (interaction between *Bias* and *Tasks*: t-statistic = 5.70, *p* < 10^−7^).

In the AMNT task, *follow* and *against* slopes did not significantly deviate from zero (AMNT-*against*: CI = [−3.14, 11.55]; AMNT-*follow*: CI = [−12.82, 0.93]) whereas costs and benefits of MTs in the PROB task showed clear asymmetry (PROB-*against*: CI = [55.92, 107.2]; PROB-*follow*: CI = [−17.82, −1.03]).

**Figure 6:**
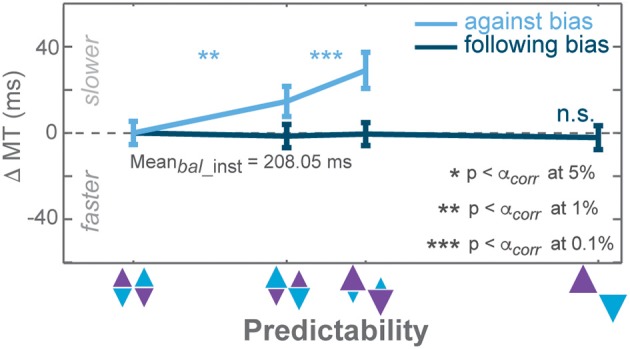
**Biasing effects on movement times (MT) instructed *against* trials of the PROB task**. Zero line indicates average MTs in instructed trials of zero-bias condition. Dark and light colors represent *follow* and *against* responses, respectively. Error bars depict standard errors. (^*^ *p* < α_*corr*_ at 5%, ^**^ *p* < α_*corr*_ at 1%, ^***^ *p* < α_*corr*_ at 0.1%, paired *t*-test with *Bonferroni* correction).

Notably, only MT cost in *against* trials of the PROB task showed gradual biasing effect whereas MTs remained unchanged in *follow* instructed trials in the PROB task (Figure 6), choice trials in the PROB task, and all types of trials in the AMNT task (not shown).

As previous studies showed that motor planning reduces motor variability (e.g., Harris and Wolpert, 1998; Todorov, 2004; Churchland et al., 2006), we tested as an additional confirmation of the MT result, if there was a biasing effect on endpoint variability (EPV), defined as the average distance of reach endpoints to the mean reach endpoint for each target location. In congruence with MT results, GLMM showed significantly higher EPVs of *against* reaches in the PROB task as compared to the AMNT task (interaction between *Bias* and *Tasks*: t-statistic = 2.42, *p* = 0.01).

Also consistent with the MT results, EPV only in *against* but not *follow* trials of the PROB task showed significant deviation from zero (PROB-*against*: t-statistic = 2.69, *p* < 0.01; PROB-*follow*: t-statistic = 1.87, *p* > 0.05) while only *follow* slope marginally deviated from zero in the AMNT task (AMNT-*against*: t-statistic = −0.15, *p* > 0.05; AMNT-*follow*: t-statistic = −2.29, *p* < 0.05).

In summary, only when being instructed against a bias in the PROB task, subjects showed effects on motor execution, visible by elevated MTs and EPVs with increasing bias degree. Neither instructions to go against a bias in the AMNT task nor choosing freely in any task had an effect on MTs and EPVs.

## Discussion

In our rule-based selection task with sequential cueing, we informed participants about the probability (PROB task) or reward amount (AMNT task) of a subsequent instruction. Using interspersed, equal-valued, and risk-free choice trials, we probed to what extent the *a priori* predictability or preferability of the upcoming rule induced a behavioral bias in subjects. Our results showed multiple biasing effects of movement planning due to predictability when compared to conditions that dealt with preferability of a goal but without planning being encouraged. (1) Subjects' responses were faster and less prone to errors in instructed trials when the final instruction matched the more likely or higher-valued rule (*follow* trials). (2) Responses were slower and more error-prone when the instruction matched the less likely or lower-valued rule (*against* trials). The strength of *follow* and *against* effects was in general not symmetric. (3) In the absence of an instruction, without any objective advantage, subjects more frequently chose the originally more likely rule, while this was true to a much lesser degree for the originally higher-valued rule. Subjects gained a reaction time advantage only in case of choosing the originally more likely rule, not the originally higher-valued rule. (4) Having to go against the more likely rule (but not against the higher-valued rule) slowed movement times and raised endpoint variability, while freely chosen movement execution was unaffected by prior expectations. Our results indicate a structural difference between decision biases resulting from predictability or preferability. These results are not consistent with the idea that the value-based decision process is a graded version of the probability-based decision process, as suggested earlier. Instead our results suggest that preliminary action planning is the major driving force for pending choice behavior and acts via different mechanisms than preference.

### Probing bias with neutral choice trials

Inducing bias with instructed trials and probing bias with choice trials was an important feature of our task design that revealed differences between the consequences of predictability and preferability. Previous studies have compared the effect of prior probability with the effect of reward amount (“potential pay-off”) on choice using partly ambiguous sensory evidence (Maddox and Bohil, 1998; Simen et al., 2009; Leite and Ratcliff, 2011; Mulder et al., 2012). In one study, subjects had to decide between two alternatives of a random dot motion stimulus, the probability or the reward amount of which was announced at the beginning of each trial and—different to here—guaranteed until the end of the trial (Mulder et al., 2012). Choices were risky due to the uncertainty about the evidence provided by the partly ambiguous stimulus. Results showed a weaker effect of potential payoff compared to prior probability. However, the fact that the difference in value between options was known from the start of each trial might have encouraged subjects to preliminarily plan the corresponding action, since in risky choices this would on average be advantageous. The fact that both types of prior expectations led to RT differences was taken as an indication for a shift of the DDM baseline in both cases. The reduced strength of effect for prior value expectations compared to probability expectations was accounted for by assuming an intermediate baseline shift but otherwise equivalent underlying mechanisms (Maddox and Bohil, 1998; Bogacz, 2007; Mulder et al., 2012). The competition-between-reward-and-accuracy-maximization (COBRA) hypothesis (Maddox and Bohil, 1998; Maddox, 2002) was proposed to explain the intermediate baseline shift in these perceptual decision-making experiments. In COBRA the reduced biasing effect of payoff manipulation is due to a conflict between (biased) reward and (unbiased) accuracy maximizing criteria, while both criteria show (biased) synergistic effects in case of probabilistic manipulation (Ashby et al., 1998; Maddox, 2002; Bogacz et al., 2006; Mulder et al., 2012). While the intermediate-baseline-shift model could account for the behavior observed in our instructed trials it does not predict the observed patter of CP and RT in the choice trials, as discussed in the following paragraphs.

Two features of our experimental design helped to decide whether biases induced by predictability and preferability can be accounted for by the same mechanism. First, two sequential cues provided subjects with the necessary information, with the second cue either instructing a specific rule (100% evidence) or allowing subjects to choose freely between both rules (rule-neutral evidence). In the DDM concept, the onset of the second cue (rule-cue), which also signals the subjects to immediately make a reach, marks the initiation of the drift process. Obeying causality, this implies that the rule-cue can only have an influence on the drift period whereas the baseline could only be set by the prior knowledge provided by the pre-cue. Importantly, as the free-choice cue provides no additional evidence supporting either rule, an effect of the prior information—either probability or reward amount—on the baseline should persist in the choice trials. In contrast, any effect of expectation that does not affect instructed and choice trials in the same way cannot be mediated by baseline changes but must indicate changes during the drift process following the instructive rule-cue. As a second important feature, we varied prior expectations gradually. If prior expectations on value are based on the same mechanism as prior expectations on probability (except for a scaling factor) then graded effects in one case should also result in graded effects in the other case.

By introducing reward-neutral interspersed choice trials we also avoided confounds between planning and motivation. If *a priori* expected values and later actual values are always identical, then motivational effects of the actually different rewards (Franchina and Brown, 1971; Hollerman et al., 1998; Hassani et al., 2001; Dayan and Balleine, 2002; Mir et al., 2011) cannot be disentangled from the effect of *a priori* expectations on the reward. This makes it difficult to account for potential effects of action planning and motivation when assessing the effect of *a priori* expected value compared to prior probabilities. Here we compare *a priori* preferability and predictability in their effect on equal-valued choice trials, thereby avoiding motivational confounds.

### Predictability leads to reduced migration distance

Our results from the PROB task support the view that prior probability affects migration distance in DDM. Equivalent effects of probability bias were observed in instructed and choice trials, allowing for a mechanism that starts prior to the rule-cue, i.e., during the DDM baseline period. In choice trials, in particular, as subjects always received the same reward for each possible choice, the reason that both options were not chosen equally often cannot be due to a reward difference but must result from the biasing effect of the prior probability. A reduction of the migration distance to the boundary associated with the more likely rule can well explain the ER and RT benefits observed for following the biased rule by instruction and by choice, as well as the CP shift in choice.

Further support for a reduced migration distance toward the threshold of the predicted target (biased threshold) is provided by a higher frequency of errors with fast RTs in case of instruction to go against the bias. A short migration distance implies that the threshold can be reached with small fluctuations toward the biased threshold, which comes at the cost of wrongly choosing the option associated with the closer boundary (Bogacz et al., 2010; Heitz, 2014) thus occur at shorter RTs than correctly instructed responses (Smith and Ratcliff, 2004; Brown and Heathcote, 2008).

**Figure 7:**
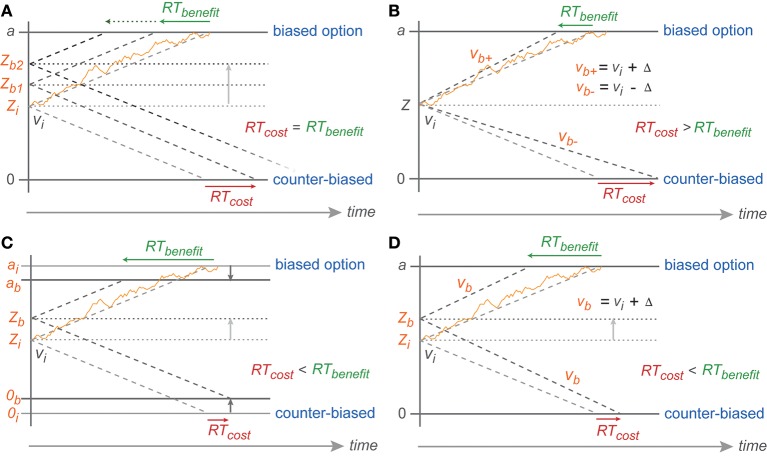
**Predicted symmetric effect of baseline shift and asymmetric effect of drift rate change**. **(A)** Baseline shift of the same magnitude should result in symmetric RT cost-benefit whereas **(B)** RT shortening toward biased reach goal due to drift rate increase (*v*_*b*+_) (from the neutral drift rate: *v*_*i*_) should be smaller than RT cost toward counter-biased reach goal due to drift rate decrease *(v*_*b*−_) of the same magnitude (Δ). Partial boundary lowering **(C)** and drift rate increase **(D)** explanations for asymmetric cost-benefit RTs in instructed reaches. *0, a, v, Z* represent counter-biased boundary, biased boundary, drift rate, and baseline, respectively. The subscript *i* indicates *initial* parameters in absence of any bias (non-biased trials) whereas subscript *b* represents parameters in case of bias with higher number depicting higher degree of bias and + vs. − depicting parameters toward biased vs. counter-biased boundaries, respectively.

However, the reduced migration difference toward the biased threshold cannot have been achieved by a pure baseline shift. With a pure baseline shift, one would expect symmetric costs and benefits, since the migration distance toward one boundary is reduced by the same amount as it is increased for the other boundary (Figure 7A). Instead, we found that the ER and RT benefits for following the bias were larger than the costs for going against the bias.

Because of the direction of the asymmetry in our data, we rule out that the biasing effects in the PROB task are explained by a bias-proportional anti-symmetric change in drift rates. By this, we mean an increased drift rate toward the biased option and decreased drift rate toward the counter-biased option, each proportional to the bias degree. In this case one would expect a cost-benefit asymmetry opposite to the asymmetry observed, i.e., larger RT costs than RT benefits (Figure 7B). This is because in the DDM the RTs are reciprocally proportional to the drift rate. Increasing drift rate hence leads to an RT benefit that is smaller in absolute value than the RT costs associated with a same-amount decrease in drift rate. This is the same logic that explains how a drift-rate which symmetrically varies around an average drift rate creates the typically observed left-skewed RT distributions. While a pure baseline shift is not sufficient to explain the behavioral results, the ER and RT data in the PROB task also cannot be explained by a pure or an additional bias-proportional anti-symmetric change in drift rate.

Rather, the asymmetry in gradual costs and benefits can be explained with models that, in addition to the baseline shift, allow (1) a bias-proportional gradually reduced migration distance by a lowered boundary for the biased option or the counter-biased option or both (Figure 7C) or (2) a bias-proportional symmetric increase in drift rate toward both options (Figure 7D).

While the behavioral consequences of predictability were largely equivalent between instructed and choice trials, there were also two differences. First, RTs were overall 20–30ms slower in choice trials, and, second, no RT costs were imposed when subjects chose freely against the biased rule. Neither difference contradicts the idea of a reduced migration distance for the biased option.

The average RT offset of 20–30 ms is not surprising since instructed trials always provided instantaneous and unambiguous evidence whereas choice trials provided rule-neutral evidence. Higher RTs for choice compared to instruction could be due to: (1) a slower drift rate due to absence of clear evidence in the choice case (Roitman and Shadlen, 2002; Hanks et al., 2015); (2) higher decision thresholds in the case of symmetric reward choice (Cavanaugh et al., 2006; Cavanagh et al., 2011; Summerfield and Tsetsos, 2012); (3) an increased duration of non-decision time, which delays migration initiation, due to unclear stimuli (Mulder and van Maanen, 2013; Coallier and Kalaska, 2014); or some combination of these possibilities. Only processes that occur with or after the rule-cue can account for differences between instructed and choice trials since subjects were unaware of the trial type prior to the rule-cue. Unless thresholds became adapted with presentation of the rule-cue, this would argue in favor of different drift rates for instructed and choice trials. As subjects had to respond within a fixed time limit and waiting longer would not have provided additional evidence, the exact amount of RT offset in choice trials is probably determined by an internal urgency signal (Cisek et al., 2009).

**Figure 8:**
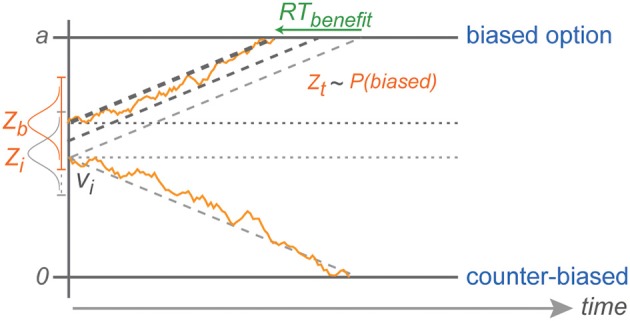
**Explanation of counter-biased choice**. Z depicts the range in which the stochastic baseline shift operates. Counter-biased choice is possible only when the baseline shift in a given trial is still close to the initial baseline (lightest gray horizontal dotted line in the middle). This explains the pattern of choice RTs against the bias that are similar to choice RTs in the zero-bias condition.

The fact that we did not find an increasing RT cost for choices against increasing bias is also consistent with the idea of a reduced migration distance and can be explained by the stochastic nature of the diffusion process in DDM (Brown and Heathcote, 2008; Heathcote and Love, 2012). With neutral evidence provided by the rule-cue, subjects will chose against the bias only when the decision variable due to random fluctuations is coincidentally around the level of the original baseline for counter-biased trials, or even closer to the counter-biased boundary at the time of the commitment to a choice. With an increasing shift in the baseline away from the initial baseline level (toward the biased boundary), *against* choices become less and less likely (explaining the CPs), but *against* choice RTs are still independent of the degree of choice bias since they always start with the same distance from the counter-biased boundary (Figure 8).

In summary, our results from the PROB task confirm the hypothesis of a reduced migration distance due to predictability. This reduction is well-explained by the combination of a bias-proportional baseline shift and either a bias-proportional reduction in threshold separation or a symmetric drift-rate increase.

### Preferability has a different effect on choice than predictability

A main question of our study was whether planning of an optional action *per se* is responsible for later choice biases. We therefore tested for differences in bias between conditions in which an action is more likely to be requested later, compared to when the same action leads to a higher potential reward in the unpredictable case that it will be requested. Our results from the instructed trials of the AMNT task fit the predictions of the intermediate-baseline-shift hypothesis discussed above, while the results from the choice trials do not.

Predictability and preferability cause structurally, not just gradually, different behavioral bias. Consistent with earlier findings (Maddox and Bohil, 1998; Simen et al., 2009; Leite and Ratcliff, 2011; Mulder et al., 2012) and the intermediate-baseline-shift hypothesis (Bogacz et al., 2006), we observed a stronger biasing effect on ER and RTs in instructed PROB trials than in instructed AMNT trials. Note that the bias degrees in our experiment were carefully matched in terms of *a priori* expected value between both tasks (see Materials and Methods). One could still expect different subjective utilities between corresponding bias degrees of both tasks, depending on the subject's level of risk aversion, e.g., devaluing the higher rewards of the AMNT task which have only a 50% chance of becoming available in the end. Yet, we do not think that differences in expected utility explain the quantitative difference in the strength of biasing effects between both task types for two reasons. First, doubling of the contrast between high and low reward {3:3, 1:5, 0.5:8.5, 0:12} in our control experiment did not alter the behavioral findings. Second, our maximal RT benefit of approximately 20 ms in the AMNT task matched the magnitude observed in a previous study with an even higher reward contrast of 20:1 (Staudinger et al., 2011). Rather, the results suggest that however high the reward ratio is, the RT costs and benefits driven by reward amount manipulation do not reach the level of RT costs and benefits observed with probability manipulation.

Our results suggest that preferability leads to drift rate changes, not to baseline changes. First, we should have observed similar patterns of biasing effects in the choice trials and the instructed trials of the AMNT task if biasing effects in the AMNT task were mediated by baseline shifts. Yet CPs were much weaker and RT differences were absent in choice trials of the AMNT task, suggesting an effect that occurs at the earliest at the appearance of the rule-cue, i.e., an effect that is independent of baseline shifts. Our results are therefore more compatible with the idea that in the AMNT task the bias-dependent RT costs and benefits are explained by drift rate adaptations that reflect the final expected reward after integration of the rule-cue. Therefore, in reward-balanced choice trials the subject's initial expectations are neutralized, leading to a lack of bias-dependent costs or benefits. The additional bias-independent fixed offset of RT between choice trials and instructed trials is of the same amount (20–30 ms) as in the PROB task, hence is likely to have the same mechanistic explanation.

Second, in the PROB task we found that RT benefits in instructed and choice trials and CPs in choice trials gradually increased with increasing levels of predictability. None of the three gradual effects was observed with increasing preference in the AMNT task. RT benefits in instructed trials and CPs in choice trials increased more or less in a single step as soon as the preferability was unbalanced, without a further increase with increasing bias degree. These observations contradict the idea that the effect of preferability is just an attenuated form of the effect of predictability.

Taken together, biasing effects due to pure motor-goal preferability are limited compared to predictability and likely restricted to processes following the final rule instruction. Once the final reward value is known after the rule instruction, adaptation of drift rate could reflect motivational effects for the immediately pending action, including shallow drift rates corresponding to demotivation when subjects were instructed to reach low- or non-rewarded targets.

### Predictability and movement planning

As we illustrated with the real-world example in the introduction, it is plausible to believe that an above-chance likelihood of later being instructed to aim for a specific goal encourages movement planning to achieve that goal. This should be the case in the biased trials of our PROB task. In contrast, chance likelihood of either goal renders preliminary movement planning toward one of the two remaining options pointless, even with varying preference for the two alternatives as in our AMNT task. This assumption was supported by our observed movement kinematics. In contrast to experiments requesting button presses (e.g., Maddox and Bohil, 1998; Mulder et al., 2012), our subjects performed extended reaches, allowing for such analysis. It was only in the PROB task, and not the AMNT task, that we found significant increase of MTs and EPVs when subjects had to go against an increasing bias. This suggests countermanding of a preliminary movement plan only in the PROB task. Based on neuronal evidence from motor planning areas, it has been proposed that when monkeys face multiple movement alternatives, the multiple candidate actions are simultaneously reflected in the movement planning activity of sensorimotor cortex preceding choice (Cisek, 2007; Scherberger and Andersen, 2007; Lindner et al., 2010; Klaes et al., 2011); In particular, a non-preferred or unselected action might not be completely suppressed before the chosen action is initiated (Cisek, 2012). Our MT and EPV results showed an effect on motor execution consistent with the idea of subjects having to disengage from a predominating motor plan in favor of a less predominating alternative plan in the PROB task only.

In summary, by dissociating preference-independent action planning (biased PROB trials) from action-independent preference (biased AMNT trials), we were able to link the processes underlying predictability with action planning within the DDM framework. According to this view, our results provide evidence that action planning modulates the migration distance in DDM, while preference modulates drift rate.

## Conclusion

Our results suggest different mechanisms underlying biasing effects of prior predictability and preferability in decision-making. This finding supports the affordance competition hypothesis (Cisek, 2007); preliminary competitive movement planning in favor of one of two potential equal-valued movement options can induce a graded choice bias and reaction time advantage, while value-based preferences alone do not.

### Conflict of interest statement

The authors declare that the research was conducted in the absence of any commercial or financial relationships that could be construed as a potential conflict of interest.
